# Vascular inflammaging: Endothelial CEACAM1 expression is upregulated by TNF‐α via independent activation of NF‐κB and β‐catenin signaling

**DOI:** 10.1111/acel.14384

**Published:** 2024-10-21

**Authors:** Lisa Götz, Uwe Rueckschloss, Andreas Reimer, Heike Bömmel, Andreas Beilhack, Süleyman Ergün, Florian Kleefeldt

**Affiliations:** ^1^ Institute of Anatomy and Cell Biology, University of Wuerzburg Wuerzburg Germany; ^2^ Department of Internal Medicine II University Hospital Wuerzburg Wuerzburg Germany; ^3^ Department of Stem Cell and Regenerative Biology and the Harvard Stem Cell Institute Harvard University Cambridge Massachusetts USA

**Keywords:** Aging, CEACAM1, endothelium, inflammation, NF‐κB, TNF‐α, β‐catenin

## Abstract

Chronic inflammation with progressive age, called inflammaging, contributes to the pathogenesis of cardiovascular diseases. Previously, we have shown increased vascular expression of the Carcinoembryonic antigen‐related cell adhesion molecule 1 (CEACAM1) in aged mice and humans, presumably via mutual upregulation with the pro‐inflammatory cytokine TNF‐α. CEACAM1 is critical for aging‐associated vascular alterations like endothelial dysfunction, fibrosis, oxidative stress, and sustained inflammation and can be regarded as a main contributor to vascular inflammaging. This study was conducted to elucidate the mechanisms underlying endothelial CEACAM1 upregulation by TNF‐α in detail. Using wildtype (WT) and TNF‐α knockout (*Tnf* ^*−*/−^) mice, we confirmed that the aging‐related upregulation of endothelial CEACAM1 critically depends on TNF‐α. The underlying mechanisms were analyzed in an endothelial cell culture model. TNF‐α time‐dependently upregulated CEACAM1 in vitro. In pharmacological experiments, we identified an early NF‐κB‐ and a delayed β‐catenin‐mediated response. Involvement of β‐catenin was further substantiated by siRNA‐mediated knockdown of the β‐catenin‐targeted transcription factor TCF4. Both signaling pathways acted independent from each other. Elucidating the delayed response, co‐immunoprecipitation analysis revealed release of β‐catenin from adherens junctions by TNF‐α. Finally, TNF‐α activated Akt kinase by increasing its Ser^473^ phosphorylation. Consequently, Akt kinase facilitated β‐catenin signaling by inhibiting its degradation via phosphorylation of GSK3β at Ser^9^ and by increased phosphorylation of β‐catenin at Ser^552^ that augments its transcriptional activity. Taken together, our study provides novel mechanistic insights into the aging‐related, inflammation‐mediated endothelial upregulation of CEACAM1. Beyond the pathogenesis of cardiovascular diseases, these findings may be significant to all fields of inflammaging.

## Graphical abstract

Early response: TNF‐α activates NF‐κB signaling by IKK‐induced degradation of the inhibitor of nuclear factor kappa B (IκB). Released NF‐κB translocates into the nucleus and upregulates CEACAM1 expression detectable after 24 h of TNF‐α stimulation. Delayed response: TNF‐α‐induced adherens junction disassembly releases β‐catenin, promoting its cytosolic and nuclear accumulation. Nuclear β‐catenin binds to transcription factors of the TCF/LEF family to induce CEACAM1 expression. Akt kinase inhibits GSK3β by phosphorylation thereby stabilizing β‐catenin and directly phosphorylates β‐catenin which was reported to increase its transcriptional activity.

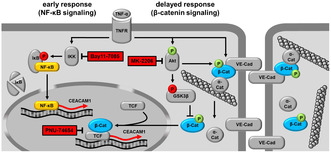

**Abbreviations**
4‐HNE: 4‐hydroxynonenal (a product of lipid peroxidation)
a.u.: arbitrary units
AJ: adherens junction
Akt: protein kinase B
ANOVA: analysis of variance
Bay11‐7085: inhibitor of NF‐κB activation
BSA: bovine serum albumin
CEACAM1: carcinoembryonic antigen‐related cell adhesion molecule 1
DAPI: 4',6‐diamidino‐2‐phenylindole
DMEM: Dulbecco's Modified Eagle's Medium
EA.hy926: endothelial cell line EA.hy926
FCS: fetal calf serum
FITC: fluorescein isothiocyanate
FLUOstar Omega: microplate reader. Product of BMG Labtech, Ortenberg, Germany
GAPDH: glyceraldehyde 3‐phosphate dehydrogenase
GSK3β: glycogen synthase kinase 3 beta
HUVEC: human umbilical vein endothelial cells
IKK: IκB kinase
IκB‐α: inhibitor of nuclear factor kappa B‐alpha
MK‐2206: inhibitor of Akt kinase
NF‐κB: nuclear factor kappa‐light‐chain‐enhancer of activated B cells
PBS: phosphate buffered saline
PMSF: phenylmethylsulfonyl fluoride
PNU‐74654: inhibitor of β‐catenin‐dependent transcription
RT‐PCR: reverse transcription polymerase chain reaction
siRNA: small interfering RNA
TCF4: transcription factor 4
*Tnf−/−*
: TNF‐alpha knockout (mice)
TNF‐α: tumor necrosis factor‐alpha
VE‐cadherin: vascular endothelial cadherin
VEGF: vascular endothelial growth factor
WT: wildtype

## 1 INTRODUCTION

Aging‐associated cardiovascular diseases like coronary artery disease, myocardial infarction, and stroke are one of the main factors limiting the health‐related quality and expectancy of life (Roth et al., 2020). Chronic low‐grade systemic inflammation with aging (inflammaging) of the vascular system is a key factor in the pathogenesis of cardiovascular diseases (Barcena et al., 2022; Ferrucci & Fabbri, 2018; Ungvari et al., 2018). Therefore, understanding the mechanisms underlying vascular inflammaging is a prerequisite for future treatments to prevent cardiovascular diseases more efficiently.

The glycoprotein Carcinoembryonic antigen‐related cell adhesion molecule 1 (CEACAM1) has been shown to be upregulated in angiogenic activated vascular endothelial cells, for example, by VEGF, contributing to morphogenesis of new vessels (Ergün et al., 2000; Gerstel et al., 2011). In recent years, we have shown that CEACAM1 is upregulated with progressive age within the murine and human vasculature. CEACAM1 in turn promotes aging‐associated vascular alterations like arterial fibrosis, enhanced oxidative stress as well as increased vascular permeability (Bömmel et al., 2017; Ghavampour et al., 2018; Kleefeldt et al., 2019). The mutual upregulation of TNF‐α and CEACAM1 with progressive age creates and maintains a chronic inflammatory milieu within the aging vascular system that might facilitate transition into vasculopathies like atherosclerosis in presence of additional risk factors (Kleefeldt et al., 2019, 2020). Therefore, CEACAM1 can be regarded as a key mediator of progressive vascular inflammaging. However, subsequent in vitro studies suggested that the TNF‐α‐dependent upregulation of endothelial CEACAM1 expression is only partially attributable to the well‐established TNF‐α/NF‐κB axis (Kleefeldt et al., 2019). Therefore, we speculated that another mechanism might also be involved. It is well known that TNF‐α increases vascular permeability by disassembly of inter‐endothelial adherens junction (AJ) complexes (Ghavampour et al., 2018; Komarova et al., 2017). We hypothesized that AJ disassembly by TNF‐α releases β‐catenin that in turn might also contribute to increased CEACAM1 expression with advancing age. This is in line with enhanced β‐catenin signaling in the aging vasculature (Kasacka et al., 2020; Kawarazaki et al., 2020; Marchand et al., 2011).

This study demonstrates that TNF‐α critically contributes to the upregulation of endothelial CEACAM1 expression with progressive age in vivo. TNF‐α‐mediated upregulation of endothelial CEACAM1 expression in vitro occurs in a biphasic manner, with an early response mediated by NF‐κB and a delayed response by β‐catenin that is released from AJ after their disassembly by TNF‐α. Furthermore, Akt kinase inactivates GSK3β thereby promoting stabilization of β‐catenin. Additionally, Akt kinase phosphorylates β‐catenin at Ser^552^ that was reported to enhance its transcriptional activity. Thus, we identified a novel mechanism that may be essential to the role of TNF‐α in inflammaging.

## 2 METHODS

### 2.1 Animal experiments

Wildtype (WT; C57BL/6J; The Jackson Laboratory, Bar Harbor, ME, USA) and C57Bl/6 mice deficient for TNF‐α (B6.129S‐Tnf^tm1Gkl^/J, short *Tnf* ^−/−^) were initially obtained from Jackson Laboratory, backcrossed to the albino C57Bl/6 background (C57BL/6‐Tyr^c‐2J^/J mice; Jackson Laboratory), and bred within the specified pathogen‐free animal facility of the Center for Experimental Molecular Medicine of Würzburg University, on a 12:12‐h dark–light cycle and fed standard chow and autoclaved drinking water ad libitum. Mice were analyzed at the age of 2 and 9 months, respectively. All experiments were carried out according to German regulations (Regierung von Unterfranken, Wuerzburg, Germany).

### 2.2 Cell culture

Endothelial EA.hy926 cells (CLS Cell Lines Service, Eppelheim, Germany) were cultured in DMEM/10% FCS (Thermo Fisher, Waltham, MA, USA; Biochrom GmbH, Berlin, Germany) at 37°C/5% CO_2_. Human umbilical vein endothelial cells (HUVEC; Cell Lines Service, Eppelheim, Germany) were cultured in Endothelial Cell Growth Medium MV (PromoCell, Heidelberg, Germany) at 37°C/5% CO_2_.

For stimulation experiments, confluent cell cultures were incubated with 50 ng/mL TNF‐α (Thermo Fisher) for indicated time periods. Bay11‐7085 (Cayman Chemical, Ann Arbor, MI, USA; 10 μM) was used to inhibit NF‐κB activation. PNU‐74654 (Absource Diagnostics GmbH, Munich, Germany; 20 μM) was used to inhibit transcriptional activity of β‐catenin. MK‐2206 (Biomol GmbH, Hamburg, Germany; 2 μM) was used to inhibit Akt kinase activity. Inhibitor treatment started 24 h prior to TNF‐α stimulation of cells.

### 2.3 siRNA‐mediated knockdown of TCF4

TCF4 protein expression was knocked down in endothelial EA.hy926 cells using siRNAs specific for the human *TCF4* gene (sc‐43525; Santa Cruz Biotechnology, Dallas, TX, USA). Unrelated siRNA served as control (control siRNA‐A, sc‐37007; Santa Cruz Biotechnology). Transfection of cells with siRNAs was carried out using siRNA Transfection Reagent and siRNA Transfection Medium according to the manufacturer's instructions (8 μL scale; Santa Cruz Biotechnology). Knockdown efficiency was monitored up to 6 days. According to the observed time course, TNF‐α stimulation (48 h) was started 2 days after application of *TCF4* siRNA.

### 2.4 Immunohistochemistry, immunofluorescence, immunoprecipitation, and immunoblotting

Immunohistochemical and immunofluorescence analyses were conducted as described previously (Ghavampour et al., 2018; Kleefeldt et al., 2019). For immunoprecipitation, confluent EA.hy926 cells were lysed with ice‐cold lysis buffer (10 mM Tris pH 7.5, 50 mM NaCl, 60 mM octyl glucoside, 1% Triton X‐100) supplemented with protease and phosphatase inhibitors (1 mM PMSF, 5 mM EDTA, 5 μg/mL aprotinin, 5 μg/mL leupeptin, 1 μg/mL pepstatin, 5 mM β‐glycerophosphate, 5 mM NaF, 3.5 mM Na orthovanadate). 500 μg of protein and 2 μg of β‐catenin antibody were co‐incubated overnight at 4°C. For control, the same amount of protein was incubated with an unrelated mouse IgG. Immunocomplexes were captured using Protein A/G UltraLink® Resin beads (Thermo Fisher) according to the manufacturer's instructions. Co‐precipitated VE‐cadherin was quantified in subsequent immunoblot analyses and was normalized to the IgG heavy chain signal. Immunoblot experiments were conducted according to a protocol described before (Ghavampour et al., 2018; Kleefeldt et al., 2019, 2022). Values for the proteins of interest were normalized to GAPDH. Primary antibodies used in this study are listed in Table 1. Secondary antibodies were purchased from Dako (Agilent Technologies, Lexington, MA, USA) and Pierce (Thermo Fisher).

**TABLE 1:** Primary antibodies used in this study.

Protein	MW (kDa)	Application	Supplier	Order No.
pS^473^‐Akt kinase	60	Immunoblotting	Cell Signaling Technology	9271
β‐catenin	92	Immunoblotting Immunofluorescence	BD Biosciences	BD‐610153
β‐catenin		Immunoprecipitation	Santa Cruz Biotechnology	sc‐7963
pS^552^‐β‐catenin	92	Immunoblotting	Cell Signaling Technology	5651
CEACAM1	160	Immunoblotting	Cell Signaling Technology	14771
CEACAM1		Immunofluorescence	Sino Biological	50571‐R030
Fibrillarin	36	Immunoblotting	Santa Cruz Biotechnology	sc‐374022
GAPDH	36	Immunoblotting	Proteintech	60004‐1‐lg
pS^9^‐GSK3β	47	Immunoblotting	Cell Signaling Technology	9323
4‐HNE		Immunohistochemistry	Bioss	BS‐6313R
IκB‐α	41	Immunoblotting	Santa Cruz Biotechnology	sc‐1643
pS^536^‐p65 NF‐κB	65	Immunoblotting	Cell Signaling Technology	3033
NF‐κB p65		Immunofluorescence	Santa Cruz Biotechnology	sc‐8008
TCF4	60	Immunoblotting	Santa Cruz Biotechnology	sc‐166699
TNF‐α		Immunohistochemistry	Santa Cruz Biotechnology	sc‐52746
β‐tubulin	55	Immunoblotting	Santa Cruz Biotechnology	sc‐58886
VE‐cadherin	130	Immunoblotting Immunofluorescence	Santa Cruz Biotechnology	sc‐9989

### 2.5 Nuclear extracts

Confluent endothelial EA.hy926 cells were harvested in ice‐cold lysis buffer (10 mM Tris pH 8.0, 5 mM CaCl_2_, 3 mM Mg acetate, 2 mM EDTA, 0.5 mM EGTA, 1 mM DTT, 0.2% Triton X‐100) containing protease and phosphatase inhibitors. Lysates were homogenized and incubated on ice for 12 min. Nuclei were pelleted (1000 × g, 5 min, 4°C) and washed three times in PBS/0.1% BSA/0.1 mM EDTA (500 × g, 5 min, 4°C) followed by serial filtration through 40 μm and 20 μm cell strainers (pluriSelect, Leipzig, Germany). Isolated nuclei were harvested in RIPA lysis buffer (Santa Cruz Biotechnology) containing protease and phosphatase inhibitors and were sonicated. Nuclear β‐catenin was quantified by immunoblotting and normalized to the nuclear marker protein fibrillarin.

### 2.6 Transwell assay

Endothelial EA.hy926 cells were grown to confluence on the apical side of 24‐well cell culture inserts (PET membrane with 1 μm pore size; Cellqart, Sabeu, Northeim, Germany) and were incubated with recombinant human TNF‐α (50 ng/mL). Fluorescein isothiocyanate‐dextran (70 kDa; Merck, Darmstadt, Germany) was added to the upper chamber to a final concentration of 1 mg/mL. Samples were collected from the lower chamber at indicated time points and fluorescence intensity was measured at 485/520 nm (excitation/emission) using a microplate reader (FLUOstar Omega, BMG Labtech, Ortenberg, Germany).

### 2.7 Picrosirius red (collagen) staining

Picrosirius red staining was performed as described previously (Vogel et al., 2015). In brief, paraffin‐embedded aortic cross‐sections were dewaxed, hydrated and stained with Weigert's hematoxylin. Subsequently, collagen fibers were stained for 1 h using 1% Direct Red 80 in a saturated aqueous solution of picric acid (both from Sigma‐Aldrich). Sections were washed twice with acidified water and were dehydrated using ethanol solutions.

### 2.8 Evans blue extravasation

Endothelial permeability was determined by means of subendothelial Evans Blue deposition as described previously (Bömmel et al., 2017).

### 2.9 Quantitative real‐time RT‐PCR

After isolation of total RNA (Qiazol; Qiagen, Hilden, Germany) and DNase I treatment (Promega, Madison, WI, USA), RNA was transcribed into cDNA (SuperScript II; Thermo Fisher). mRNA expression was analyzed by real‐time PCR in triplicate for each sample (GoTaq qPCR Master Mix; Promega). Carryover of genomic DNA was monitored by running an amplification with SuperScript II‐deficient RT reaction as template in parallel for each sample. Primers used in this study were purchased from Invitrogen (Thermo Fisher) and are listed in Table 2. After completion of the PCR cycling protocol (95°C/30 s; 60°C/30 s; 72°C/30 s; 40 cycles), the specificity of amplification was proven by melting curve analyses. Values for the mRNA of the respective genes were normalized to *GAPDH* mRNA.

**TABLE 2:** Real‐time PCR primers used in this study.

Gene	Gene bank Acc. No.	Upstream primer (5′ → 3′)	Downstream primer (5′ → 3′)
*CEACAM1*	NM_001712.4	GAAAATGGCCTCTCACCTGGGG	CTATCAGAGCAACCAGGGCCAC
*CCND1*	NM_053056.3	CACCGACAACTCCATCCGGC	TGGGGCTCCTCAGGTTCAGG
*AXIN2*	NM_004655.4	GGAGATGACCCCCGTGGAAC	GCGGGTCTTCCTCGTAGCTG
*MYC*	NM_002467.6	ACAACACCCGAGCAAGGACG	AACGTTGAGGGGCATCGTCG
*GAPDH*	NM_002046.7	TGACAACTTTGGTATCGTGGA	CCAGTAGAGGCAGGGATGAT

### 2.10 Statistical analyses

All experiments were repeated at least three times with similar results. Data are given as arbitrary units (a.u.) and are presented as mean ± SEM. Statistical significance was tested using a two‐tailed Student's t test (two groups) or analysis of variance (ANOVA, multiple groups). The null hypothesis was rejected at *p* < 0.05 (*).

## 3 RESULTS

### 3.1 Upregulation of endothelial CEACAM1 expression with increasing age by TNF‐α in vivo

First, we sought to validate our in vivo model. To this end, we compared thoracic aortae from 2 months and 9 months old mice. We found that endothelial permeability as determined by Evans Blue extravasation increased from 2 to 9 months. Similarly, the content of collagen fibers within the vascular media as detected by Picrosirius Red staining increased. Finally, immunohistochemical analyses demonstrated higher TNF‐α expression in the aortae of 9 months old mice (Figure S1).

**FIGURE 1:**
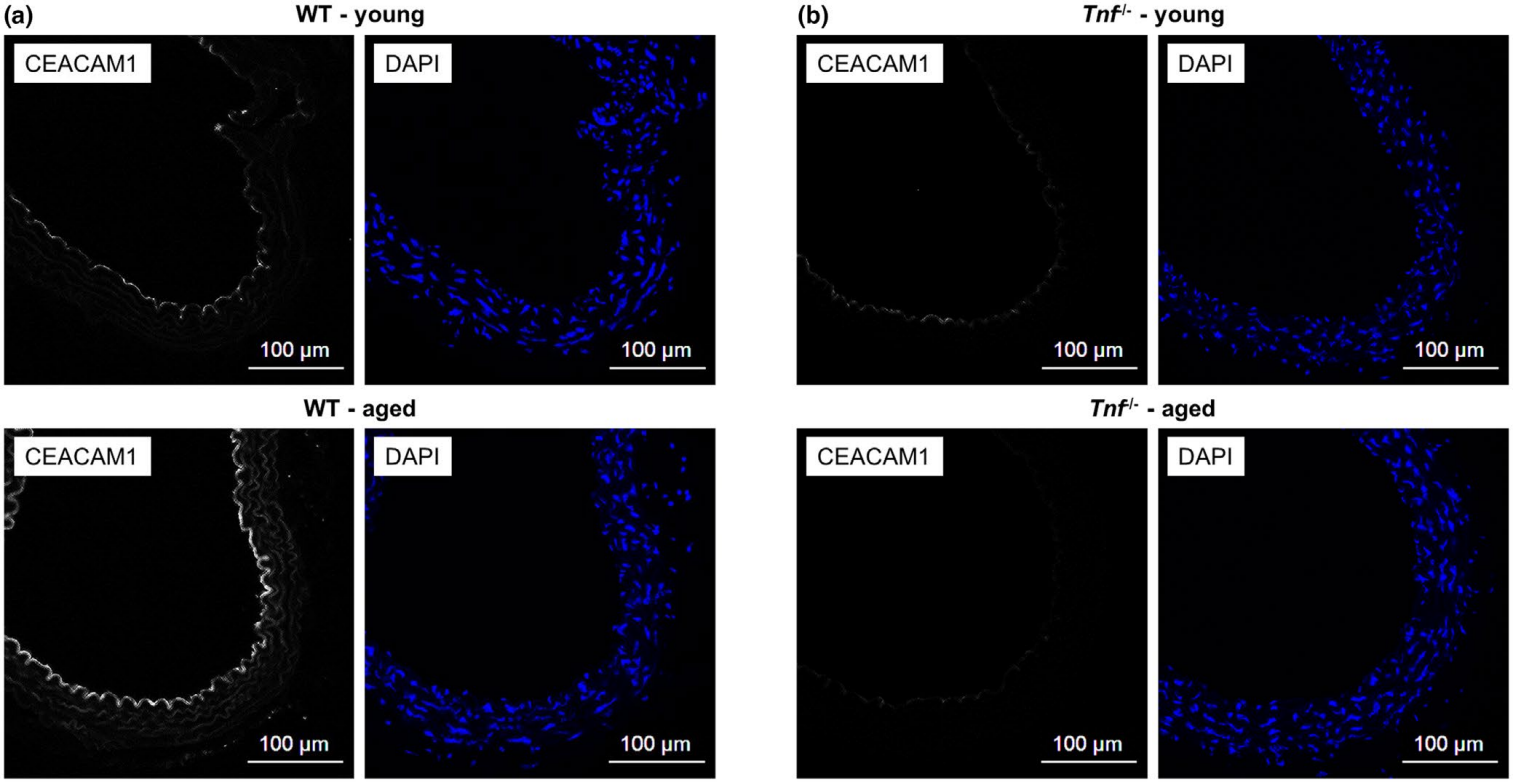
Aging‐associated upregulation of endothelial CEACAM1 expression by TNF‐α in vivo. CEACAM1 immunostainings of cross‐sections from thoracic aortae of young (2 months) and aged (9 months) mice. Nuclei were visualized using DAPI. (a) CEACAM1 is predominantly expressed within the endothelium and increases with advancing age in WT mice. (b) In contrast, this age‐dependent increase is absent in *Tnf* ^−/−^ mice. Representative micrographs from *N* = 3 independent experiments.

Based on our previous findings we hypothesized that aging‐related upregulation of vascular CEACAM1 expression is TNF‐α‐dependent (Kleefeldt et al., 2019). Therefore, we performed CEACAM1 immunofluorescent analyses using cross‐sections of thoracic aortae from WT and *Tnf* ^−/−^ mice. Endothelial CEACAM1 expression increased from 2 months to 9 months of age in WT mice (Figure 1a). However, a similar increase in endothelial CEACAM1 immunoreactivity with increasing age was absent in *Tnf* ^−/−^ mice (Figure 1b).

### 3.2 Time‐dependent upregulation of endothelial CEACAM1 expression by TNF‐α via activation of NF‐κB and β‐catenin signaling in vitro

**FIGURE 2:**
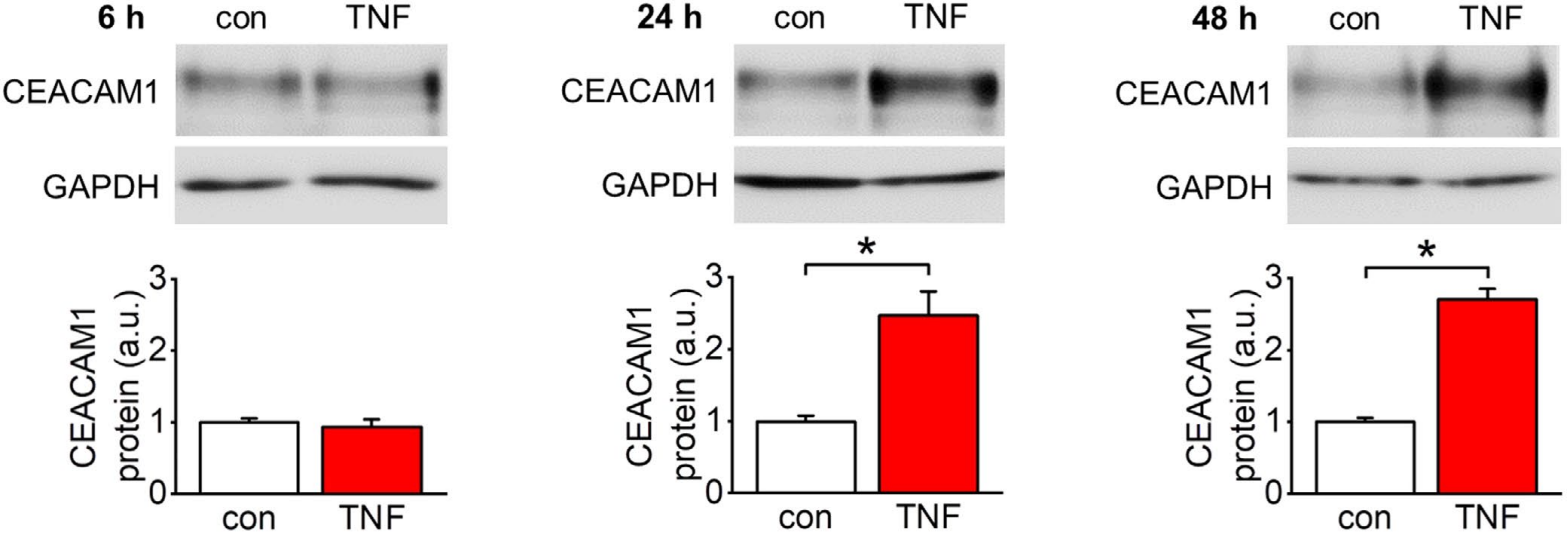
Time‐dependent induction of CEACAM1 expression by TNF‐α in vitro. Quantification of CEACAM1 protein expression in endothelial cells (EA.hy926). Cells were either untreated (control) or stimulated with TNF‐α (50 ng/mL) for indicated time periods. TNF‐α significantly upregulated CEACAM1 expression after 24 h and 48 h of incubation. *n* = 5–6; *p < 0.05.

**FIGURE 3:**
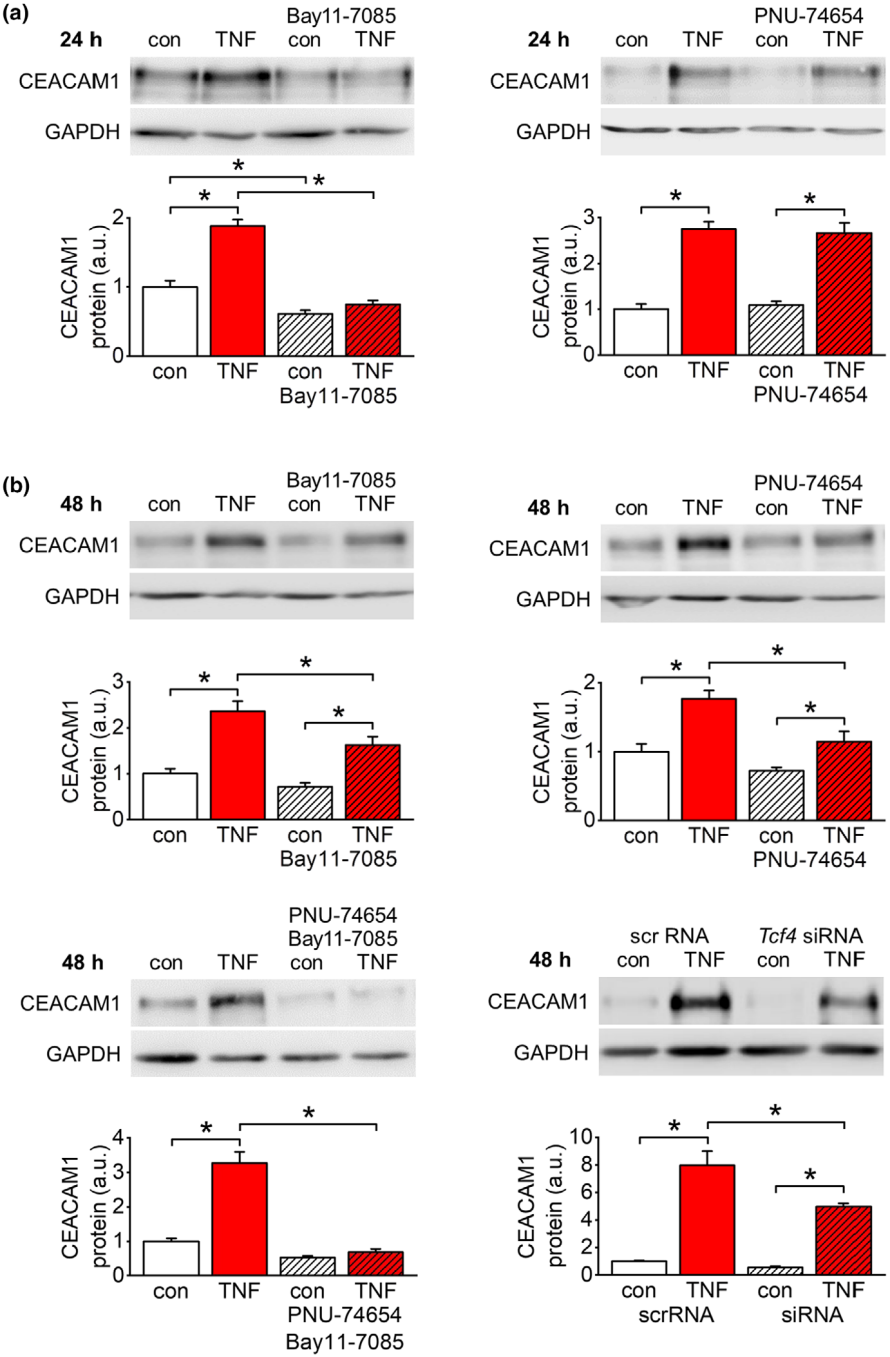
CEACAM1 upregulation by early and delayed responses to TNF‐α stimulation. (a) Pharmacological inhibition of CEACAM1 upregulation in EA.hy926 cells at 24 h of TNF‐α stimulation (50 ng/mL). TNF‐α‐dependent induction of CEACAM1 expression in EA.hy926 cells is completely blocked by the NF‐κB signaling inhibitor Bay11‐7085 (10 μM). In contrast, application of the β‐catenin signaling inhibitor PNU‐74654 (20 μM) shows no effect on CEACAM1 upregulation at that time point. These results indicate a decisive role of NF‐κB signaling in this early response. *n* = 5–6; *p < 0.05. (b) Pharmacological/genetic inhibition of CEACAM1 upregulation in EA.hy926 cells at 48 h of TNF‐α stimulation (50 ng/mL). Separate application of each inhibitor partially attenuates induction of CEACAM1 expression at 48 h of TNF‐α stimulation. However, concomitant application of both inhibitors completely blocks TNFα‐dependent upregulation of CEACAM1 expression indicating an independent contribution of both signaling pathways to this delayed response. Attenuation of TNF‐α‐dependent CEACAM1 upregulation by siRNA‐mediated knockdown of the β‐catenin‐targeted transcription factor TCF4 further supports involvement of β‐catenin signaling. *n* = 4–6 (except siRNA experiment: *n* = 3); *p < 0.05.

The TNF‐α‐dependent upregulation of CEACAM1 expression was elucidated in more detail using the endothelial cell line EA.hy926. CEACAM1 protein expression significantly increased after TNF‐α stimulation of cultured endothelial cells for 24 h and 48 h, whereas no effect could be observed after 6 h (Figure 2). A more chronic stimulation of EA.hy926 cells (5 ng/mL, on day 1, 3, and 5 of a 7‐days period) similarly upregulated CEACAM1 expression (Figure S2). Specific pharmacologic inhibitors were used to identify signaling pathways involved in TNF‐α‐dependent CEACAM1 upregulation. Application of Bay11‐7085 (NF‐κB signaling inhibitor; 10 μM) completely blocked the increase in CEACAM1 expression by TNF‐α at 24 h (Figure 3a) but was only partially effective at 48 h (Figure 3b). In contrast, application of PNU‐74654 (inhibitor of β‐catenin‐dependent transcription; 20 μM) showed no effect at 24 h (Figure 3a) but partially prevented TNF‐α‐induced CEACAM1 expression at 48 h (Figure 3b). Interestingly, concomitant application of Bay11‐7085 and PNU‐74654 completely blocked CEACAM1 upregulation by TNF‐α at 48 h (Figure 3b).

In addition, using specific siRNA we suppressed protein expression of TCF4, a transcription factor targeted by nuclear β‐catenin. This intervention reduced TCF4 levels by approximately 65% compared to control cells transfected with scrambled RNA (Figure S3A). This TCF4 knockdown attenuated TNF‐α‐induced CEACAM1 expression to a similar extend as pharmacological inhibition of β‐catenin signaling using PNU‐74654 (Figure 3b).

Finally, TNF‐α stimulation of endothelial cells enhanced transcription of the well‐known β‐catenin target genes *CCND1*, *AXIN2*, and *MYC* (Figure S3B).

### 3.3 TNF‐α‐induced *CEACAM1* transcription independent from de novo protein synthesis

**FIGURE 4:**
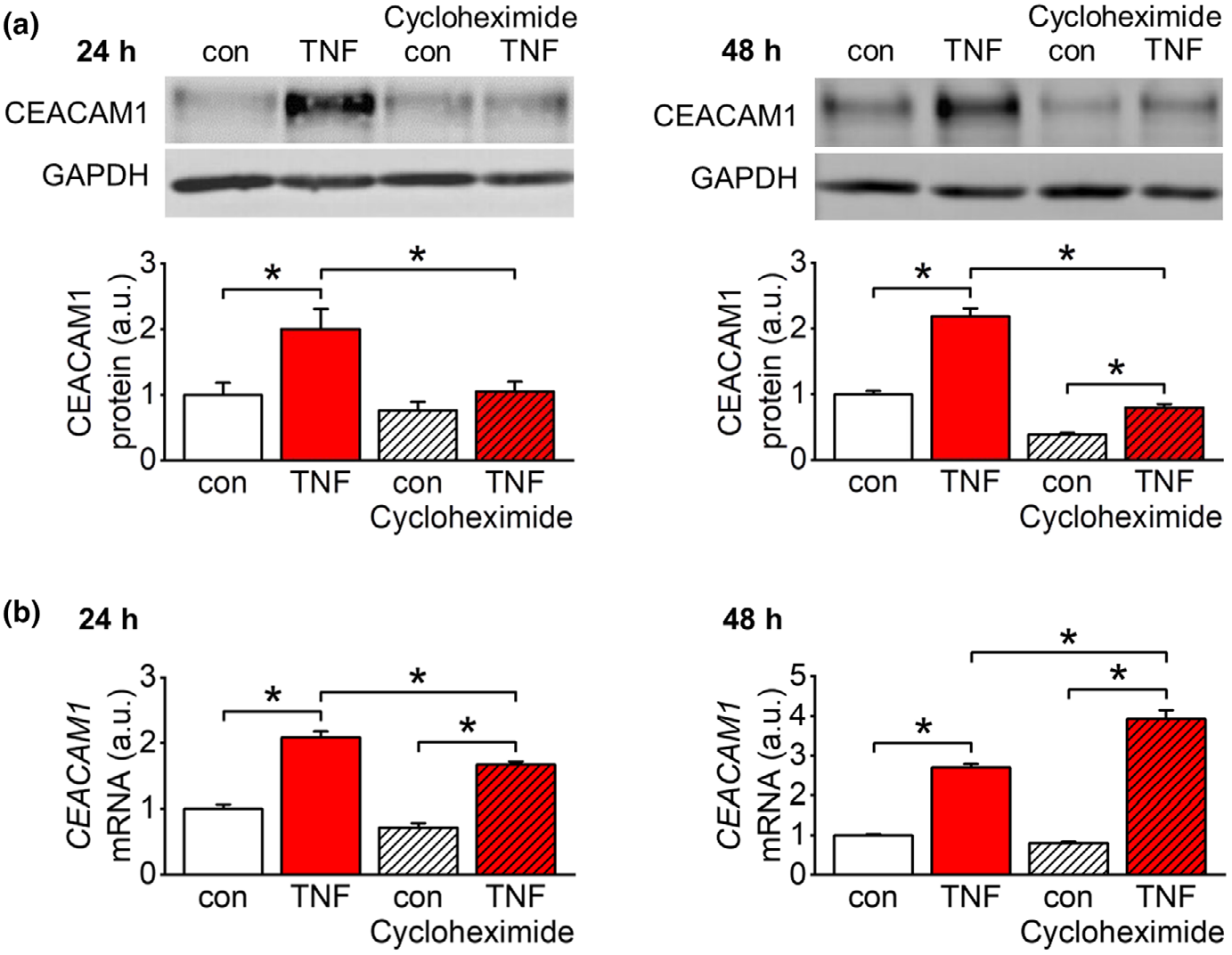
Induction of *CEACAM1* transcription by TNF‐α independent from de novo protein synthesis. (a) Quantification of CEACAM1 protein expression after TNF‐α stimulation (50 ng/mL) in the presence of the protein translation inhibitor cycloheximide (100 nM) in EA.hy926 cells. At both time points, upregulation of CEACAM1 protein level is almost completely blocked by cycloheximide indicating efficient inhibition of de novo protein synthesis. *n* = 5–6; *p < 0.05. (b) Quantification of *CEACAM1* mRNA transcription after TNF‐α stimulation (50 ng/mL) in the presence of cycloheximide (100 nM) in EA.hy926 cells by real‐time RT‐PCR. TNF‐α‐induced *CEACAM1* mRNA transcription is preserved even if de novo protein synthesis is inhibited by cycloheximide excluding the contribution of another transcription factor downstream of NF‐κB or β‐catenin. *n* = 4–6; *p < 0.05.

To further characterize how NF‐κB and β‐catenin affect *CEACAM1* transcription, we analyzed the TNF‐α‐dependent effect in the presence of the protein translation inhibitor cycloheximide (100 nM). Complete inhibition of CEACAM1 protein upregulation after 24 h and 48 h of TNF‐α stimulation in the presence of cycloheximide proved an efficient block of de novo protein synthesis (Figure 4a). However, TNF‐α‐dependent induction of *CEACAM1* transcription was largely preserved at both time points in the presence of cycloheximide (Figure 4b).

### 3.4 Release of β‐catenin from adherens junctions by TNF‐α and nuclear translocation

**FIGURE 5:**
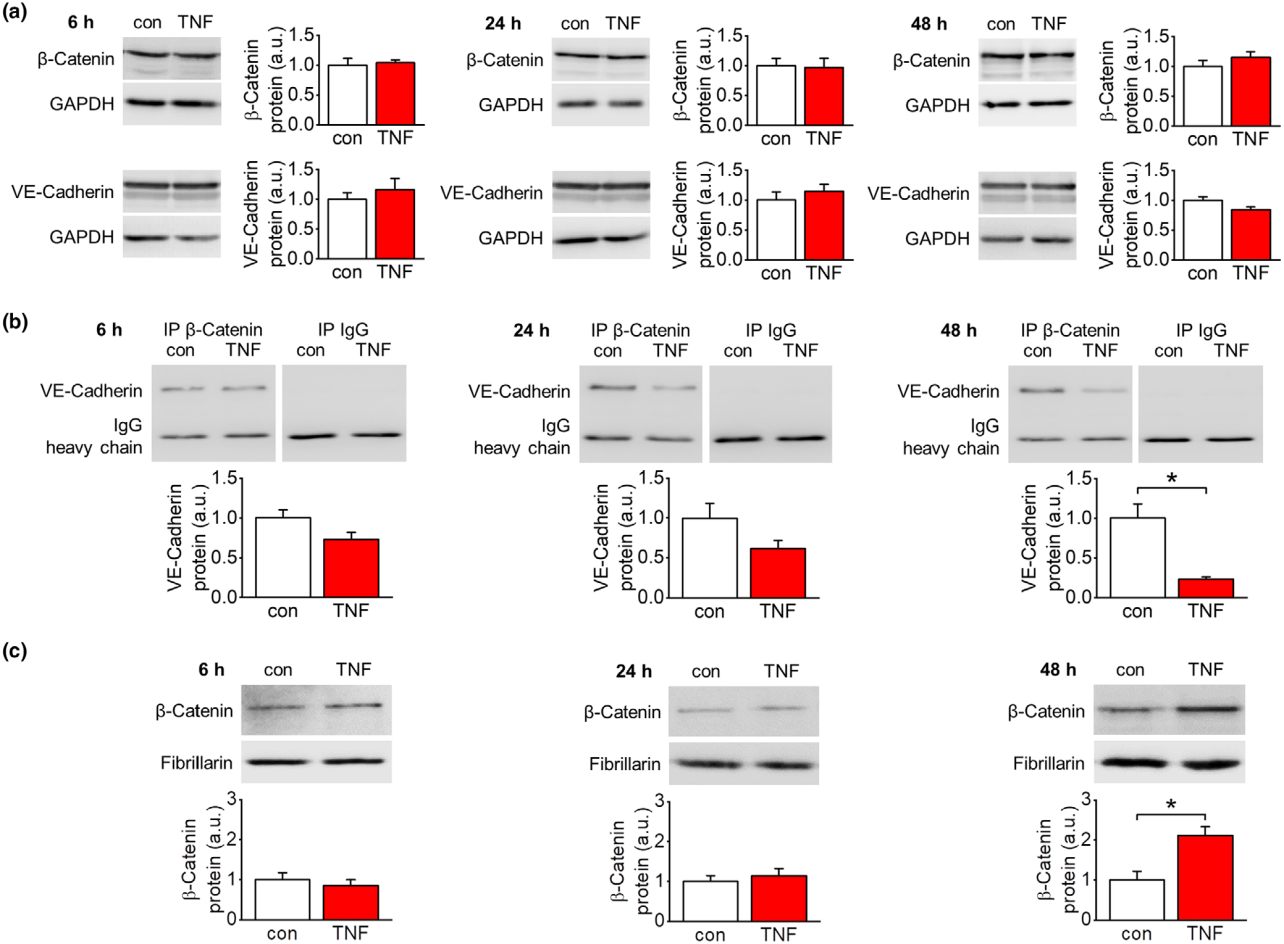
Release of β‐catenin from AJs by TNF‐α and nuclear translocation. (a) Quantification of β‐catenin and VE‐cadherin protein expression after TNF‐α stimulation. (50 ng/mL) of EA.hy926 cells. TNF‐α does not affect expression of both proteins. *n* = 5–6. (b) Analysis of β‐catenin/VE‐cadherin interaction in response to TNF‐α stimulation (50 ng/mL) of EA.hy926 cells by immunoprecipitation. The amount of VE‐cadherin that is co‐precipitated with β‐catenin decreased over time gaining statistical significance only after 48 h. *n* = 3; *p < 0.05. (c) Quantification of β‐catenin in nuclear extracts of EA.hy926 cells after TNF‐α stimulation (50 ng/mL). In accordance with the time course of its release from AJs, nuclear accumulation of β‐catenin is only detectable after 48 h. *n* = 3; *p < 0.05.

TNF‐α treatment of endothelial cells did not alter protein expression of the AJ proteins VE‐cadherin and β‐catenin (Figure 5a).

TNF‐α‐dependent alterations in the interaction of VE‐cadherin and β‐catenin within endothelial AJs were analyzed by co‐immunoprecipitation experiments. AJ complexes were precipitated using an anti‐β‐catenin antibody. Within the precipitate, the amount of VE‐cadherin protein decreased over time. This decline gained statistical significance after 48 h of TNF‐α stimulation (Figure 5b). Accordingly, permeability of endothelial EA.hy926 monolayers to FITC‐labeled dextran (70 kDa) significantly increased only after 48 h and 72 h of TNF‐α stimulation (Figure S4).

We also analyzed nuclear translocation of β‐catenin. To this end, we generated nuclear extracts of untreated or TNF‐α‐stimulated endothelial cells (Figure S5). According to the pharmacological data, nuclear β‐catenin protein levels increased only after 48 h of TNF‐α treatment (Figure 5c).

### 3.5 TNF‐α‐dependent phosphorylation of Akt kinase, GSK3β, and β‐catenin

**FIGURE 6:**
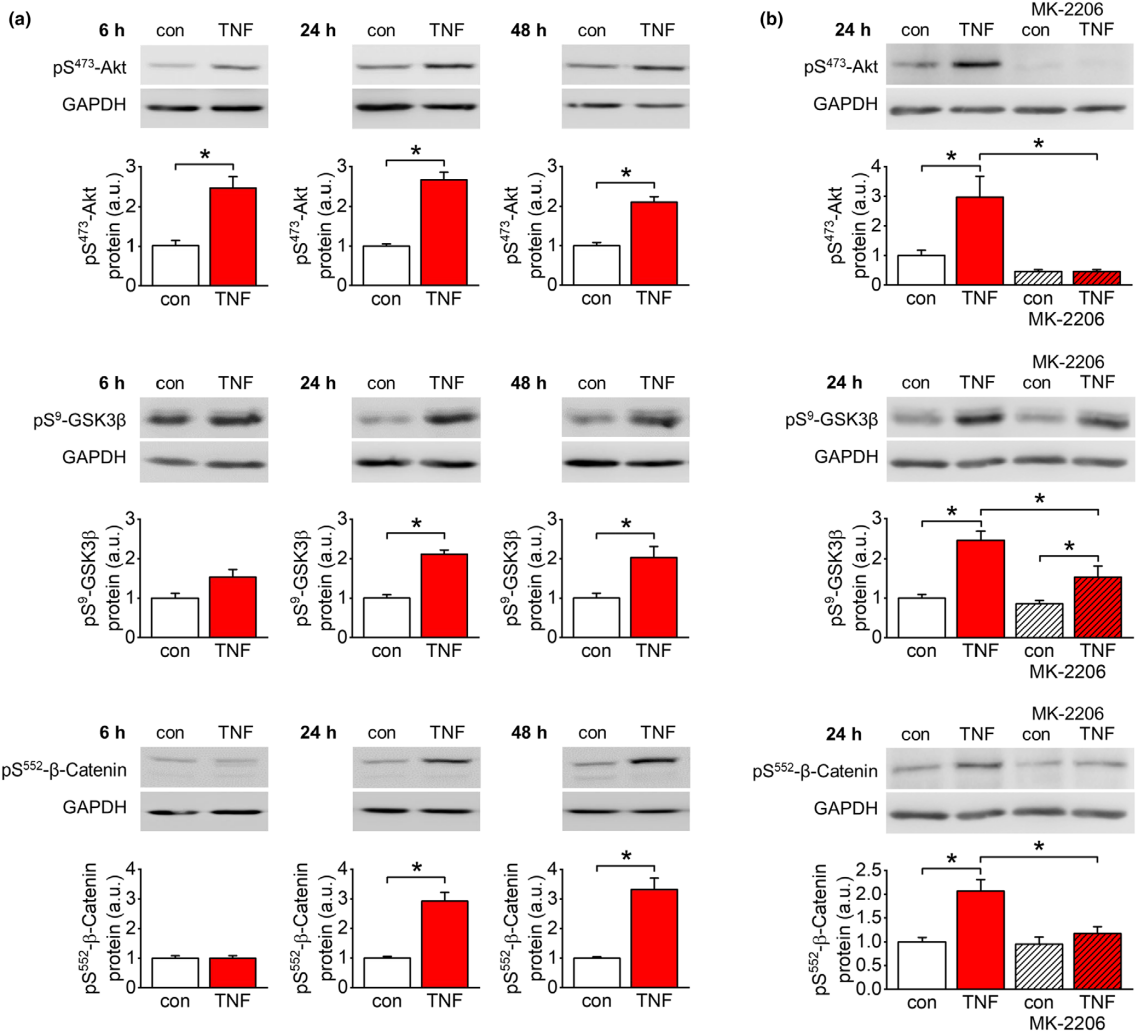
TNF‐α‐dependent phosphorylation of Akt kinase, GSK3β, and β‐catenin. (a) Quantification of pS^473^‐Akt kinase, pS^9^‐GSK3β, and pS^552^‐β‐catenin in response to TNF‐α stimulation (50 ng/mL) of EA.hy926 cells for different periods. TNF‐α induced activating phosphorylation of Akt kinase at all time points analyzed. The inhibiting phosphorylation of GSK3β and the activity‐promoting phosphorylation of β‐catenin significantly increased after 24 h and 48 h of TNF‐α stimulation. *n* = 4–6; *p < 0.05. (b) Quantification of pS^473^‐Akt kinase, pS^9^‐GSK3β, and pS^552^‐β‐catenin after 24 h of TNF‐α stimulation (50 ng/mL) in the presence of the Akt kinase inhibitor MK‐2206 (2 μM) in EA.hy926 cells. All three phosphorylation sites are at least partially sensitive to Akt kinase inhibition. *n* = 4–5; *p < 0.05.

At all incubation periods analyzed, TNF‐α increased phosphorylation of Akt kinase at Ser^473^ in endothelial cells (Figure 6a). Similarly, TNF‐α also augmented phosphorylation of GSK3β at Ser^9^ (Figure 6a) and of β‐catenin at Ser^552^ at 24 h and 48 h of stimulation (Figure 6a). Inhibition of Akt kinase activity by MK‐2206 (1 μM) attenuated phosphorylation of Akt kinase, GSK3β, and β‐catenin at least partially after 24 h of TNF‐α stimulation (Figure 6b).

### 3.6 Lack of crosstalk between NF‐κB and β‐catenin signaling pathways

**FIGURE 7:**
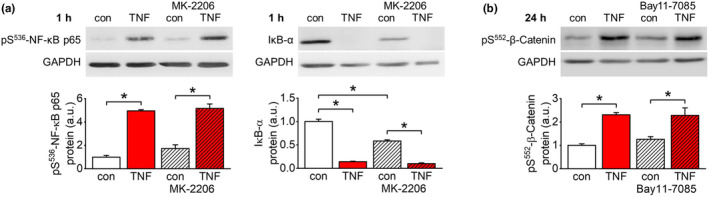
Lack of crosstalk between NF‐κB and β‐catenin signaling. (a) Quantification of pS^536^‐p65 NF‐κB and IκB‐α in response to TNF‐α stimulation (50 ng/mL) of EA.hy926 cells for 1 h. TNF‐α induces phosphorylation of the NF‐κB subunit p65 as well as degradation of IκB‐α, both indicating activation of NF‐κB signaling. These alterations are not abolished in the presence of MK‐2206 (2 μM), an inhibitor of Akt kinase that is involved in β‐catenin activation. *n* = 4–5; *p < 0.05. (b) Quantification of pS^552^‐β‐catenin in response to TNF‐α stimulation (50 ng/mL) of EA.hy926 cells for 24 h. TNF‐α‐induced phosphorylation of β‐catenin is insensitive to inhibition of NF‐κB signaling using Bay11‐7085 (10 μM). *n* = 4–5; *p < 0.05.

TNF‐α stimulation of endothelial cells for 1 h increased phosphorylation of NF‐κB at Ser^536^ and reduced the amount of IκB (Figure 7a). However, inhibition of Akt kinase activity by MK‐2206 (1 μM) did neither prevent NF‐κB phosphorylation nor IκB degradation in response to TNF‐α (Figure 7a). Vice versa, TNF‐α‐dependent phosphorylation of β‐catenin at Ser^552^ was unaffected by the NF‐κB signaling pathway inhibitor Bay11‐7085 (10 μM; Figure 7b).

### 3.7 Verification of key findings with primary endothelial cells

Immortalized cell lines may behave different from primary cells in certain aspects of their biology. Therefore, we sought to verify our main results obtained with the endothelial cell line EA.hy926 using human umbilical vein endothelial cells (HUVEC).

**FIGURE 8:**
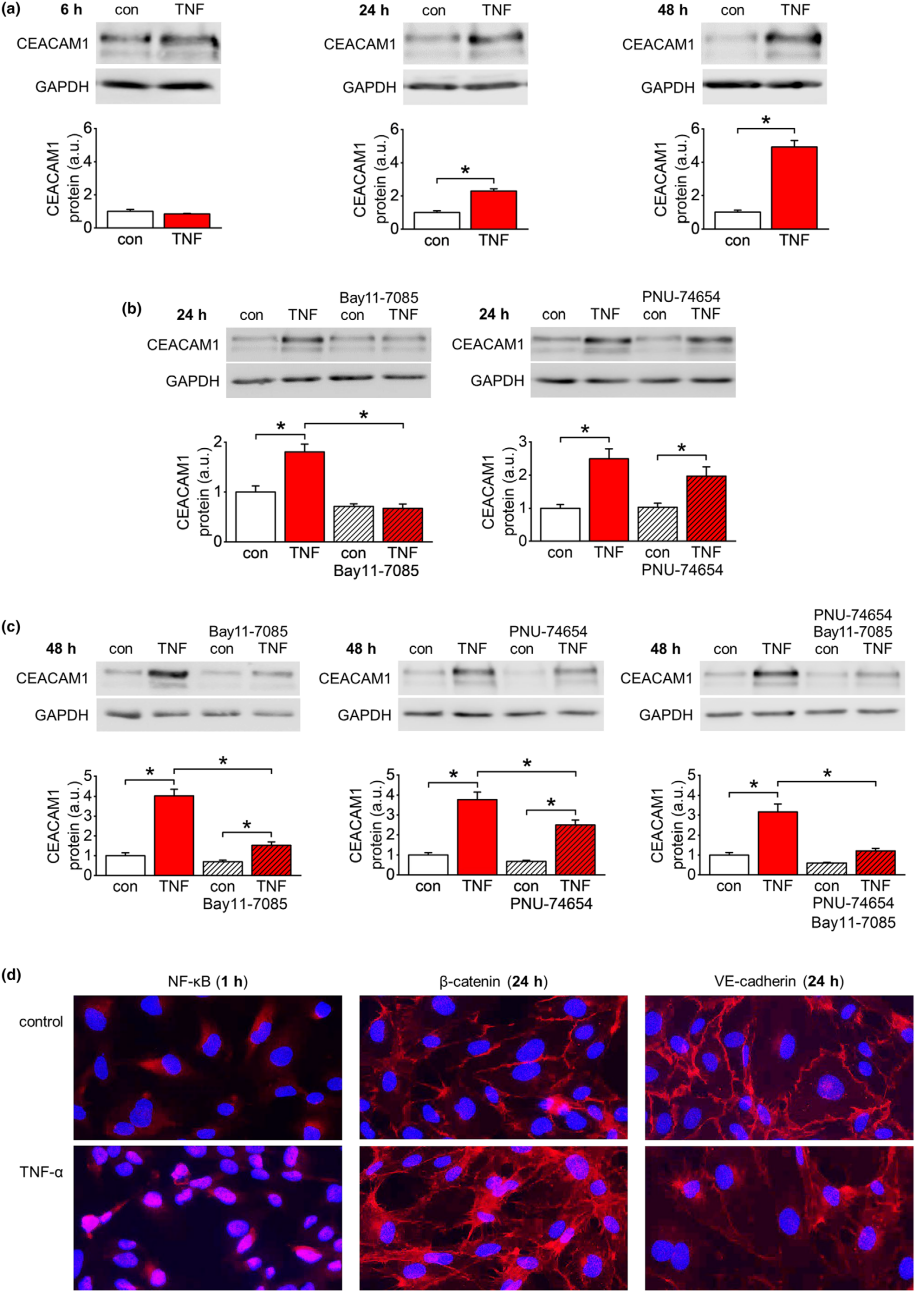
Verification of key findings in primary endothelial cells. (a) Quantification of CEACAM1 protein expression in human umbilical vein endothelial cells (HUVEC). Stimulation of cells with TNF‐α (50 ng/mL) significantly upregulated CEACAM1 expression after 24 h and 48 h of incubation. *n* = 4–5; *p < 0.05. (b) Pharmacological inhibition of CEACAM1 upregulation in HUVEC at 24 h of TNF‐α stimulation (50 ng/mL). TNF‐α‐dependent induction of CEACAM1 expression is completely blocked by the NF‐κB signaling inhibitor Bay11‐7085 (10 μM). In contrast, the β‐catenin signaling inhibitor PNU‐74654 (20 μM) does not significantly interfere with TNF‐α‐induced CEACAM1 upregulation at that time point. *n* = 4–5; *p < 0.05. (c) Pharmacological inhibition of CEACAM1 upregulation in HUVEC at 48 h of TNF‐α stimulation (50 ng/mL). Applied separately, each inhibitor partially reduces the significant CEACAM1 upregulation at 48 h of TNF‐α stimulation. However, applied concomitantly both inhibitors synergistically prevent a significant TNF‐α‐dependent upregulation of CEACAM1. *n* = 4–5; *p < 0.05. Thus, TNF‐α‐dependent upregulation of CEACAM1 shows the same time‐dependency and pharmacological characteristics in HUVEC and EA.hy926 cells. (d) Representative immunofluorescent images of control and TNF‐α‐treated HUVEC. In untreated cells, NF‐κB is localized to the cytosol. After TNF‐α stimulation (50 ng/mL; 1 h), NF‐κB is present in almost all nuclei. Under control conditions, β‐catenin, and VE‐cadherin are confined to the cell borders. In TNF‐α‐treated cells (50 ng/mL; 24 h), this clear staining pattern is completely lost in the case of β‐catenin and less pronounced regarding VE‐cadherin.

TNF‐α stimulation of confluent HUVEC cultures (50 ng/mL) time‐dependently upregulated CEACAM1 expression (Figure 8a). Furthermore, the NF‐κB pathway inhibitor Bay11‐7085 (10 μM) completely blocked TNF‐α‐dependent CEACAM1 upregulation after 24 h of incubation (Figure 8b). In contrast, the β‐catenin pathway inhibitor PNU‐74654 (20 μM) was without significant effect on TNF‐α‐induced CEACAM1 upregulation at that time point (Figure 8b). When applied separately, each inhibitor partially reduced the significant CEACAM1 upregulation at 48 h of TNF‐α stimulation (Figure 8c). However, concomitant application of both inhibitors prevented the significant TNF‐α effect on CEACAM1 expression (Figure 8c).

In addition, we conducted immunofluorescence analyses of NF‐κB, β‐catenin, and VE‐cadherin in control and TNF‐α‐treated HUVEC (50 ng/mL). As expected, NF‐κB was mainly localized to the cytosolic compartment under control conditions. However, TNF‐α stimulation (1 h) induced nuclear translocation of the transcription factor (Figure 8d). Similarly, the AJ proteins β‐catenin and VE‐cadherin that are confined to the cell membrane under control conditions were completely (β‐catenin) or partially (VE‐cadherin) redistributed to the cytosol after TNF‐α stimulation of HUVEC (24 h; Figure 8d).

## 4 DISCUSSION

In this study, we identified a novel mechanism of TNF‐α/CEACAM1 interaction in endothelial cells that may be essential to the role of TNF‐α and CEACAM1 in inflammaging. We show for the first time that (a) TNF‐α critically contributes to the upregulation of endothelial CEACAM1 expression with progressive age in vivo, (b) TNF‐α upregulates endothelial CEACAM1 expression in vitro in a biphasic manner, with an early response mediated by NF‐κB and a delayed response by β‐catenin that is released from AJ after their disassembly by TNF‐α, (c) Akt kinase inactivates GSK3β thereby promoting stabilization of β‐catenin. Additionally, Akt kinase phosphorylates β‐catenin at Ser^552^ that was reported to enhance its transcriptional activity.

Persistent low‐grade systemic inflammation with progressive age (inflammaging) is a main driver of cardiovascular pathologies. However, underlying mechanisms establishing this chronic pro‐inflammatory milieu are still inadequately understood (Ajoolabady et al., 2023; Barcena et al., 2022).

TNF‐α is a central pro‐inflammatory mediator. It has been known for a long time that the level of this cytokine is elevated in serum as well as in the vasculature of aging humans and rodents (Csiszar et al., 2003; Paolisso et al., 1998; Spaulding et al., 1997). In the present study, we confirmed increasing vascular expression of TNF‐α in mice growing from 2 months to 9 months of age. Furthermore, TNF‐α was shown to critically contribute to vascular aging by several groups (Arenas et al., 2006; Csiszar et al., 2007; Park et al., 2015). Interestingly, this aging related increase in vascular TNF‐α expression might depend on CEACAM1, since vascular TNF‐α expression is greatly reduced in older *Ceacam1* ^−/−^ mice compared to age‐matched WT mice (Kleefeldt et al., 2019). Similar to TNF‐α, vascular expression of CEACAM1 is also augmented with progressive age in mice and humans (Kleefeldt et al., 2019). This is of special importance because CEACAM1 in turn critically promotes vascular alterations like enhanced endothelial permeability and collagen deposition that are hallmarks of vascular aging (Kleefeldt et al., 2019). Furthermore, in the present study we demonstrate that these widely accepted age‐related vascular alterations are already detectable in aortae of 9‐months‐old mice thereby validating our in vivo model.

Since TNF‐α induced CEACAM1 expression in cultured endothelial cells, we hypothesized that this is also the mechanism of aging‐related upregulation of vascular CEACAM1 expression in vivo (Kleefeldt et al., 2019). This mutual upregulation of TNF‐α and CEACAM1 would establish a vicious cycle promoting inflammaging and eventually the development of cardiovascular diseases (Kleefeldt et al., 2019, 2020). Therefore, this study was conducted to identify the TNF‐α‐activated signaling pathways involved in vascular CEACAM1 upregulation.

To verify this hypothesis, we first analyzed the significance of TNF‐α for aging‐related vascular CEACAM1 upregulation in vivo. Age‐related upregulation of aortic endothelial CEACAM1 expression was readily detectable in WT mice but was absent in *Tnf* ^−/−^. This finding clearly demonstrates that upregulation of CEACAM1 within the aging vasculature critically depends on TNF‐α in vivo.

To elucidate the involved signaling pathways in more detail, further in vitro analyses were performed using an endothelial cell line. Similar to a long‐term stimulation, incubation of endothelial cells with TNF‐α time‐dependently upregulated CEACAM1 after 24 h and 48 h, respectively. Pharmacological inhibitor studies revealed that the early response entirely depends on NF‐κB activation. This is of particular interest since NF‐κB is a main mediator of inflammaging (Barcena et al., 2022; Ferrucci & Fabbri, 2018; García‐García et al., 2021; Songkiatisak et al., 2022). However, at 48 h of TNF‐α stimulation NF‐κB inhibition was only partially effective. This is in accordance with our previous findings after 72 h of TNF‐α stimulation and suggests the contribution of another signaling pathway to this delayed response (Kleefeldt et al., 2019, 2020).

TNF‐α is known to impair endothelial barrier function via disassembly of inter‐endothelial contacts, namely AJs (Ghavampour et al., 2018; Gong et al., 2014). These complexes are composed of VE‐cadherin, a transmembrane protein binding homophilic to VE‐cadherin on adjacent endothelial cells. Intracellular, VE‐cadherin is connected to the actin cytoskeleton via α‐, β‐ and p120‐catenins (Giannotta et al., 2013; Lampugnani et al., 2018). β‐catenin released by AJ disassembly might induce gene transcription as a co‐activator of the TCF/LEF transcription factor family upon nuclear translocation (Beckers et al., 2008; Masszi et al., 2004; Valenta et al., 2012). Furthermore, previous studies reported enhanced β‐catenin signaling in aging human arteries and hearts (Kasacka et al., 2020; Marchand et al., 2011). Therefore, we speculated about the contribution of β‐catenin transcriptional activity to the delayed response in TNF‐α‐mediated upregulation of CEACAM1 expression. Pharmacological inhibition of β‐catenin/TCF4 interaction (Trosset et al., 2006) was partially effective at 48 h of TNF‐α stimulation pointing to the involvement of β‐catenin transcriptional activity. Interestingly, inhibition of β‐catenin signaling did not affect CEACAM1 upregulation at 24 h of TNF‐α stimulation further supporting the decisive role of NF‐κB signaling at that earlier time point. In order to provide additional evidence for the involvement of β‐catenin signaling besides chemical inhibitor experiments, we used siRNA‐mediated knockdown of TCF4, a transcription factor mediating β‐catenin‐dependent transcription (Schuijers et al., 2014). This approach attenuated TNF‐α‐mediated CEACAM1 upregulation to a similar extent as pharmacological inhibition of β‐catenin signaling. In conjunction with the induced transcription of well‐known β‐catenin target genes, these results prove the contribution of TNF‐α‐induced β‐catenin signaling to the delayed response of CEACAM1 upregulation.

It is known that NF‐κB and β‐catenin signaling may crosstalk with each other in several ways (for review see (Ma & Hottiger, 2016)). However, concomitant pharmacological inhibition of NF‐κB and β‐catenin signaling completely prevented the delayed response to TNF‐α at 48 h. This additive effect proves an independent contribution of both pathways to TNF‐α‐mediated CEACAM1 upregulation. This conclusion is further substantiated by our finding that pharmacological inhibition of either of the two signaling pathway does not prevent TNF‐α‐dependent activation of the other one.

Next, we investigated whether NF‐κB and β‐catenin act directly on the *CEACAM1* promoter or indirectly via inducing expression of a downstream transcription factor. Since the latter option would require de novo protein synthesis, we applied TNF‐α in the presence of the protein translation inhibitor cycloheximide. Even in this experimental setting *CEACAM1* mRNA expression was still induced by TNF‐α treatment. Our data suggest that both NF‐κB and β‐catenin/TCF4 activate *CEACAM1* transcription in response to TNF‐α independent from a de novo synthesized downstream transcription factor.

The induction of NF‐κB‐dependent transcription by TNF‐α is a well‐established mechanism. In our experimental setup, activation of NF‐κB signaling pathway is evident from the TNF‐α‐induced degradation of the inhibitor of NF‐κB, IκB, and the nuclear translocation of NF‐κB. Furthermore, in response to TNF‐α stimulation we detected phosphorylation of NF‐κB at Ser^536^, a modification that was shown to increase its transcriptional activity (Sakurai et al., 2003; Zhong et al., 2002).

To characterize the β‐catenin‐based mechanism of CEACAM1 upregulation by TNF‐α in more detail, we speculated about the origin of the transcriptionally active β‐catenin. As β‐catenin protein levels were unchanged by TNF‐α stimulation and de novo protein synthesis was proven to be dispensable, we speculated about subcellular redistribution of β‐catenin, namely nuclear translocation of β‐catenin released from AJs disassembled by TNF‐α (Ghavampour et al., 2018). AJ disassembly, monitored by β‐catenin/VE‐cadherin co‐immunoprecipitation reached statistical significance only after 48 h of TNF‐α stimulation. This rather slow time course of TNF‐α‐mediated AJ disassembly in our in vitro model is further supported by our analysis of endothelial permeability to 70 kDa dextran in response to TNF‐α. Finally, we also determined the time course of β‐catenin nuclear translocation in TNF‐α‐stimulated cells. To our knowledge, this is the first analysis of TNF‐α‐induced nuclear accumulation of β‐catenin in a quantifiable manner in endothelial cells. Again, nuclear β‐catenin accumulation gained statistical significance only after 48 h of TNF‐α stimulation. Thus, both AJ disassembly and nuclear translocation are in accordance with a contribution of released β‐catenin to the delayed response.

Once released from AJs, stability of cytosolic β‐catenin is tightly controlled by the GSK3β‐containing so‐called destruction complex to prevent unregulated nuclear translocation and induction of gene transcription (Liu et al., 2022; Yost et al., 1996). GSK3β‐dependent phosphorylation of β‐catenin marks it for ubiquitylation and subsequent proteasomal degradation (Aberle et al., 1997; Park et al., 2020). Hence, release of β‐catenin from AJs alone might not be sufficient to support β‐catenin‐dependent gene transcription in response to TNF‐α. GSK3β activity is predominantly regulated by Akt kinase. Phosphorylation of GSK3β at Ser^9^ inhibits its activity thereby promoting β‐catenin stability (Cross et al., 1995; Manning & Toker, 2017). This is of particular interest since Ser^9^ phosphorylation of GSK3β was suggested to promote cardiac aging (Chen et al., 2021).

In our endothelial cell culture model, TNF‐α enhanced phosphorylation of Akt kinase at Ser^473^ from the earliest time point analyzed. This is in accordance with earlier reports on TNF‐α‐dependent Akt kinase activation (Pincheira et al., 2008; Zhou et al., 2008). Consequently, activated Akt kinase enhanced phosphorylation of GSK3β at Ser^9^, protecting released cytosolic β‐catenin from degradation and supporting its nuclear translocation and *CEACAM1* transcription. Additionally, we found that Akt kinase also directly phosphorylated β‐catenin at Ser^552^, a modification reported to promote release of β‐catenin from AJs with subsequent cytosolic and nuclear accumulation and thereby to increase its transcriptional activity (Fang et al., 2007). Thus, these two Akt kinase‐dependent phosphorylation events further support the β‐catenin‐mediated upregulation of CEACAM1 in response to TNF‐α stimulation.

We analyzed the signaling events that underly TNF‐α‐dependent upregulation of CEACAM1 expression in an immortalized endothelial cell line in detail. However, the significance of our results and conclusions is not restricted to cell lines since we confirmed main findings in primary human umbilical vein endothelial cells (HUVEC), that is, time‐dependency and pharmacological characteristics of TNF‐α‐dependent CEACAM1 upregulation.

Taken together, this study deciphers one side of the vicious cycle based on TNF‐α and CEACAM1 expression that may crucially contribute to vascular inflammaging. We show for the first time that the central pro‐inflammatory cytokine TNF‐α upregulates endothelial expression of the aging‐promoting CEACAM1 in a β‐catenin‐dependent manner. This is in line with previous reports showing a prominent role of β‐catenin signaling also in renal and muscular aging processes (Brack et al., 2007; Franzin et al., 2020). Whether this also involves upregulation of CEACAM1 expression in these organs has not been analyzed so far. Considering the impact of CEACAM1 expression on aging‐related vascular alterations (Kleefeldt et al., 2019, 2020), our present data underscore the importance of inflammation to vascular aging. Thus, long‐term global inhibition of inflammation might prevent or even slow down aging processes. However, based on adverse effects like enhanced susceptibility to infection by long‐term inhibition, neither TNF‐α nor NF‐κB are suitable pharmacological targets to decrease inflammaging (Ha et al., 2022; Leone et al., 2023). The same applies to β‐catenin due to involvement in adult tissue homeostasis and adaption (Liu et al., 2022).

Nevertheless, this study has some limitations: since the usage of HUVEC primary cells was restricted to six passages, not all experiments conducted in the EA.hy926 cell line could be performed in HUVECs as well. Furthermore, we decided to use immunohistochemical/immunofluorescent analyses due to the limited availability of *in vivo* material. In future studies, the aspects of vascular aging should be addressed in a more quantifiable manner, that is, by Western blotting.

However, based on our recent findings, CEACAM1 seems to be a more promising target in order to limit vascular inflammaging and thereby its transition into vascular pathologies. Beyond vascular inflammaging, our results on the interrelation of TNF‐α and CEACAM1 may also be of importance for nonvascular diseases like cancer progression since CEACAM1 is also involved in these diseases (Götz et al., 2023).

## AUTHOR CONTRIBUTIONS

Design of experiments, analysis and interpretation of data, manuscript preparation and review (LG, UR, SE, FK); conducting experiments (LG, UR, AR, HB, AB); supervision and grant acquisition (AB, FK).

## FUNDING INFORMATION

This study was supported by the German Research Foundation (DFG, project number: 502291066; awarded to F.K.), the Vogel Foundation Dr. Eckernkamp/“Universitätsbund Würzburg”, Würzburg, Germany (Research Advancement Award to F.K.), and German Research Foundation (DFG TRR221 B11, 324392634 to A.B.). This publication was supported by the Open Access Publication Fund of the University of Wuerzburg.

## CONFLICT OF INTEREST STATEMENT

None declared.

## Supporting information


Figure S1.

Figure S2.

Figure S3.

Figure S4.

Figure S5.

## Data Availability

Data available on request from the authors.
