# Supplementary figures and images for: Vascular inflammaging: Endothelial CEACAM1 expression is upregulated by TNF‐α via independent activation of NF‐κB and β‐catenin signaling
Source: Aging Cell. 2024 Oct 21;24(2):e14384. doi: 10.1111/acel.14384 (PMC11822634; doi:10.1111/acel.14384)

Supplement Fig. 1

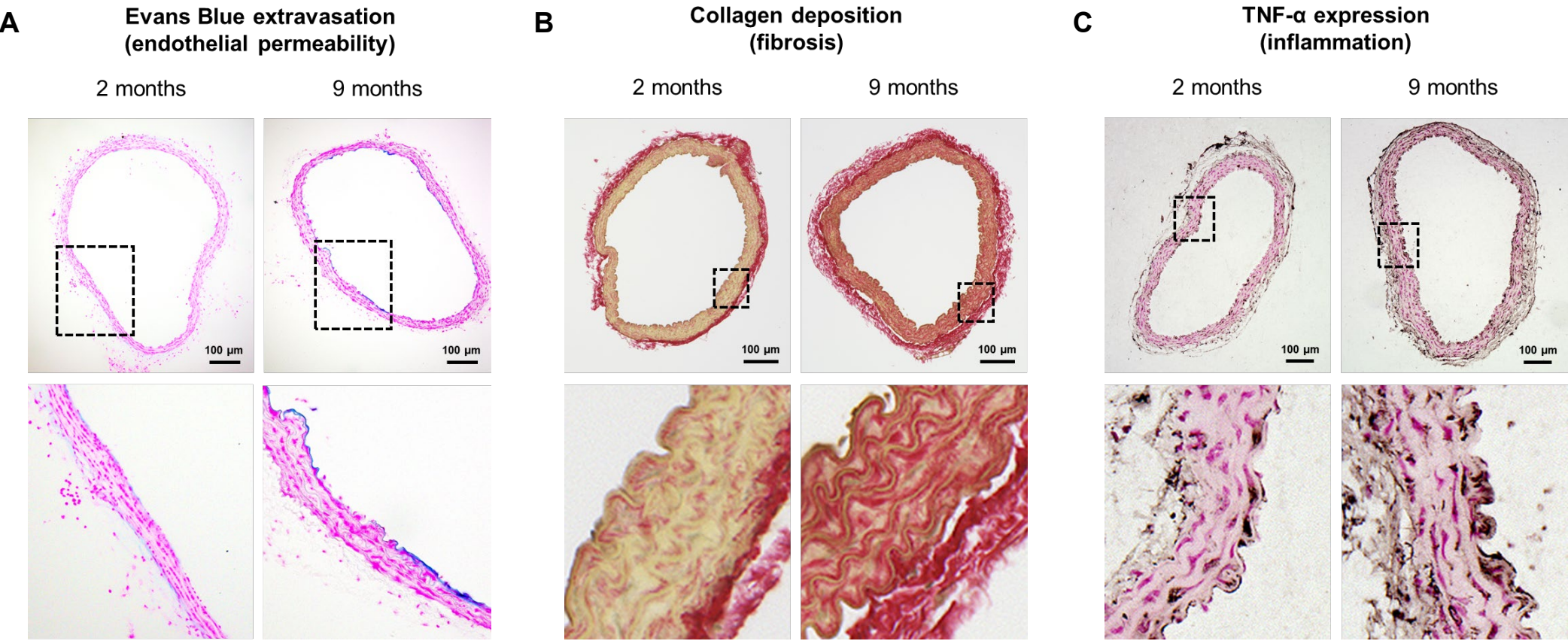

Supplement: Supplementary file 1 — Figure S1. [file ACEL-24-e14384-s004.pdf]

Supplement Fig. 2

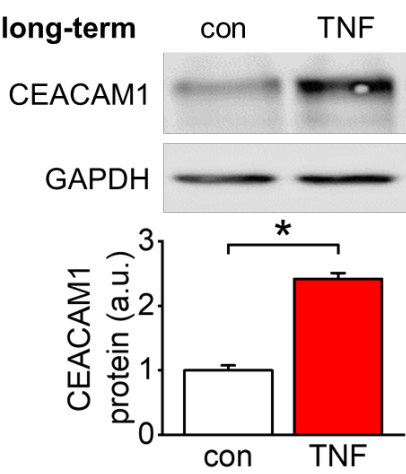

Supplement: Supplementary file 2 — Figure S2. [file ACEL-24-e14384-s005.pdf]

Supplement Fig. 3

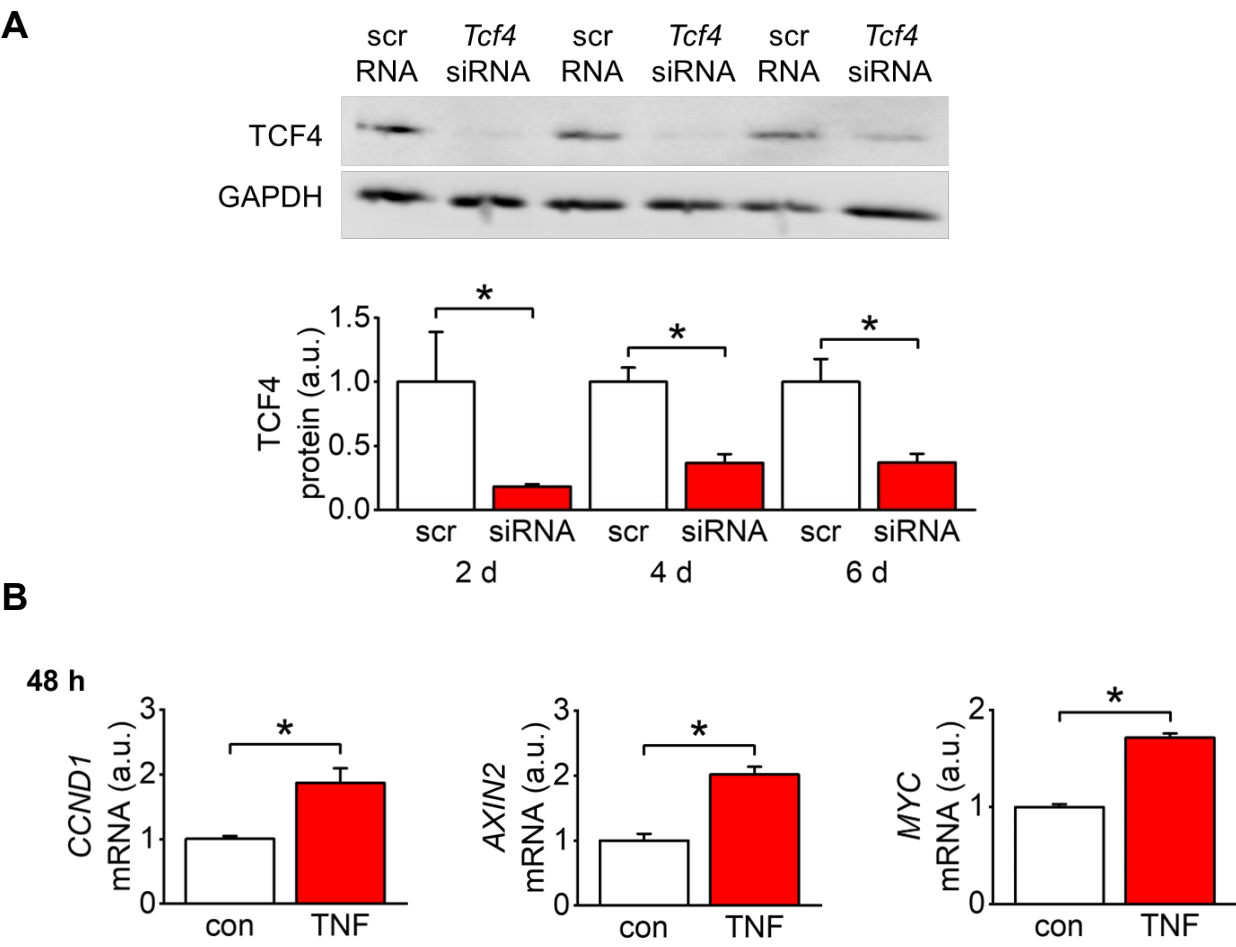

Supplement: Supplementary file 3 — Figure S3. [file ACEL-24-e14384-s001.pdf]

Supplement Fig. 4

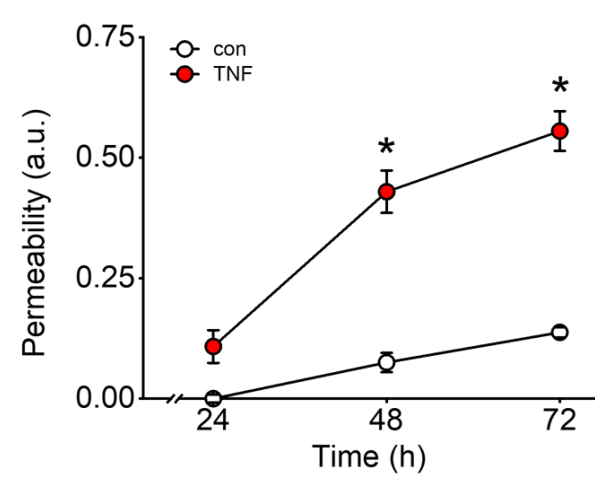

Supplement: Supplementary file 4 — Figure S4. [file ACEL-24-e14384-s003.pdf]

Supplement Fig. 5

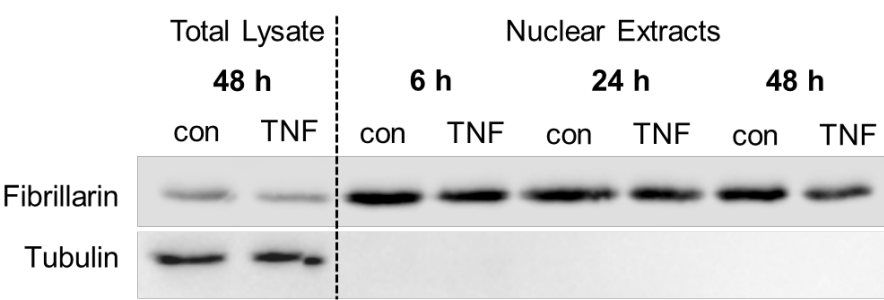

Supplement: Supplementary file 5 — Figure S5. [file ACEL-24-e14384-s002.pdf]
